# Cellulases: From Bioactivity to a Variety of Industrial Applications

**DOI:** 10.3390/biomimetics6030044

**Published:** 2021-07-05

**Authors:** Uroosa Ejaz, Muhammad Sohail, Abdelaziz Ghanemi

**Affiliations:** 1 Department of Microbiology, University of Karachi, Karachi 75270, Pakistan; uroosaejaz24@gmail.com; 2 Department of Biosciences, Shaheed Zulfikar Ali Bhutto Institute of Science and Technology (SZABIST), Karachi 75600, Pakistan; 3 Department of Molecular Medicine, Faculty of Medicine, Laval University, Quebec, QC G1V 0A6, Canada

**Keywords:** cellulase, food processing, food production, food industry

## Abstract

Utilization of microbial enzymes has been widely reported for centuries, but the commercial use of enzymes has been recently adopted. Particularly, cellulases have been utilized in various commercial sectors including agriculture, brewing, laundry, pulp and paper and textile industry. Cellulases of microbial origin have shown their potential application in various commercial sectors including textile, pulp and paper, laundry, brewing, agriculture and biofuel. Cellulases have diversified applications in the food industry, food service, food supply and its preservation. Indeed, cellulases can tenderize fruits, clarify the fruit juices, reduce roughage in dough, hydrolyze the roasted coffee, extract tea polyphenols and essential oils from olives and can increase aroma and taste in food items. However, their role in food industries has by and large remained neglected. The use of immobilized cellulases has further expanded their application in fruit and vegetable processing as it potentiates the catalytic power and reduces the cost of process. Technological and scientific developments will further expand their potential usage in the food industry.

## 1. Cellulase

Enzymes are biological molecules that are produced by all living beings. These are generally termed as “biocatalysts”. Recent trends of replacing hazardous chemicals in the industrial sector with green chemistry approaches have rendered an ever-increasing demand for enzymes. In this context, the availability of cost-effective substrates of the enzymes is very critical to render the large-scale production of chemicals feasible. Plant cell walls contain polysaccharides which can serve as energy and carbon sources that can be utilized by microorganisms, therefore, the plant cell wall can be taken as a central component of the carbon cycle. Hence, researchers around the world are involved in elucidation of the structural characteristics of the plant cell wall polysaccharides and have diverted their attention to characterize the enzymes involved in the degradation of plant cell wall polysaccharides and their encoding genes. Microbial enzymes involved in the degradation of plant cell wall convert these polysaccharides into digestible components. The sugars and the substances which are released as a result of these degradative processes serve as nutrients for the microorganism itself and for plants, and animals. The potential of these enzymes has been investigated for their commercial exploitation. Indeed, the growing field of microbial enzyme technology has resulted in development of novel enzyme-based processes [1]. Microbial enzymes are more stable than animal and plant enzymes. They offer many advantages as these can be produced with reduced cost and less space in shorter duration by fermentation techniques in high consistency; moreover, optimization of the process can be done easily [2]. The plant cell wall degrading microbial enzymes can be used for the production of human and animal foods, textiles, paper, detergents and biofuels [3]. Plant cell wall degrading enzymes including pectinases, xylanases and cellulases are among the leading enzymes produced commercially for their diversified applications. Particularly, cellulases are responsible for cellulose degradation by hydrolyzing the β-1,4-glycosidic bonds [4]. Cellulases are one of the widely used industrial enzymes which are commercially available for more than 30 years [5]. These are inducible enzymes synthesized by a various microorganism including, bacteria and fungi, during their growth on cellulosic materials. Consequently, cellulose is converted to simple sugar, glucose, which can be fermented into cellulosic biofuels [6]. 

## 2. Structure of Cellulase

Cellobiohydrolases and endocellulases consist of a signal peptide that mediates secretion, a hinge region which is rich in Pro, Thr and Ser residues, a cellulose binding domain, and a catalytic domain. N- and O-glycosylated proteins are present in catalytic domain and hinge region, respectively [7]. The bacterial and fungal cellulases usually consist of two or more functional and structural domains which are connected by a peptide linker [8]. In aerobic organisms, cellulose binding domain binds to a catalytic domain whereas, dockerin domain joins to the catalytic domain in anaerobic organisms [9]. Fungal cellulases consist of cellulose binding module (CBM) and a catalytic domain (CD); CBM is connected through a short polylinker region [5]. In fungal cellulases, catalytic binding domain is comprised of less than 40 amino acid residues which also includes three conserved aromatic residues [7]. According to the International Union of Biochemistry and Molecular Biology Enzyme Nomenclature, bacterial cellulases are grouped into EC 3.2.1.4 and are included in fourteen Glycosil Hydrolase (GH) families. Higher growth rates and genetic versatility of bacteria, emphasize the advantages and suitability of bacterial cellulases over fungal sources [10], although many fungal cellulases are commercially available. 

## 3. Mode of Action of Cellulase

Cellulase consists of three enzymes: β-glucosidase, endo-1,4-β-D-glucanase (endoglucanase) and exo-1,4-β-D-glucanase (exoglucanase). These three enzymes are involved in the hydrolysis of cellulose by synergetic action for accomplished and effective hydrolysis of cellulose [7]. The most studied cellulolytic fungus, *Trichoderma reesei*, produces seven β-glucosidases, eight endo-β-1, 4-glucanase components and two cellobiohydrolase components [8]. Endoglucanase acts on inner sites of oligosaccharides found in carboxymethyl cellulose, cellooligosaccharides or amorphous cellulose. Exoglucanase hydrolyzes non-reducing ends of crystalline cellulose and forms cellobiose or glucose as the major end products. β-glucosidase acts on non-reducing ends of cellobiose and cellodextrin [9]. 

The most common cellulolytic bacteria are *Thermobifida fusca*, *Thermonospora* sp., *Streptomyces* sp., *Ruminococcus albus*, *Thermobispora bispora*, *Erwinia chrysanthemi*, *Clostridium* sp., *Cellulomonas* sp., *Bacillus* sp., and *Acetivibrio cellulolyticus* [11]. Yet the research for the isolation and screening of new and novel cellulolytic bacteria is ever growing field. During 2012 to 2015 time span, at least 9 novel cellulolytic bacterial species which includes *Pseudomonas coleopterorum* [12], *Herbinix hemicellulosilytica* [13], *Caldicellulosiruptor changbaiensis* [14], *Anaerobacterium chartisolvens* [15], *Alicyclobacillus cellulosilyticus* [16], *Bacteroides luti* [17], *Ornatilinea apprima* [18], *Kallotenue papyrolyticum* [19], and *Streptomyces abietis* [20] were reported in the *International Journal of Systematic and Evolutionary Microbiology*. The reports on cellulolytic species in other journals are nonetheless substantially higher in number. For example, Ejaz et al. [21] recently reported the cellulolytic activity of *Bacillus aestuarii*. Huang et al. [22] isolated 207 strains of facultatively aerobic and anaerobic cellulolytic bacteria from the gut of *Holotrichia parallela* larvae. The cellulolytic potential of *Pseudomonas nitroreducens*, *Shinella zoogloeoides*, *Ensifer adhaerens*, *Labrys neptuniae*, *Devosia riboflavina*, *Kaistia adipata*, *Ochrobactrum haematophilum*, *Ochrobactrum cytisi*, *Paracoccus sulfuroxidans*, *Cellulosimicrobium funkei* and *Siphonobacter aquaeclarae* were first reported by Huang et al. [22]. Several fungal species e.g., *Aspergillus terrus* MS105 [23], *Aspergillus fumigatus* MS16 [24], *Humicola insolens* MTCC 1433 [25], *Trichoderma citrinoviride* AUKAR04 [26], *Dipodascaceae* and *Phaffomycetaceae* [27] have been reported for cellulase production.

## 4. Thermostable Cellulase

Cellulases can be obtained by either solid state or submerged fermentation from bacteria and fungi [28,29]. Being inducible enzymes, their production depends on activation and repression mechanism [30]. Cellulases are reportedly more stable at high temperatures than other plant cell wall degrading enzymes [31] which makes them a better choice for industrial applications. Therefore, we have seen extensive efforts to isolate cellulolytic microorganisms [32] and to understand the rules governing thermostability of their proteins. De Farias and Bonato [33], observed that amino acids such as Gln, Cys, Ala and His are commonly present in proteins of mesophiles, whereas, Lys, Ile, Tyr and Gly are predominately found in thermophilic microorganism’s proteins [34]. Furthermore, it is reported that salt bridges also contribute in thermostability of enzyme. Salt bridges are formed by the electrostatic interaction among Lys, Glu and Arg [35]. 

The bacterial cellulase offers many advantages compared to fungal cellulase such as facilitated mass transfer, increased specific activity and more stability [36,37,38]. The most commonly studied thermotolerant or thermophilic cellulolytic bacteria are *Thermobifida fusca*, *Thermonospora* spp., *Ruminococcus albus*, *Thermobispora bispara*, *Erwinia chrysanthemi*, *Clostridium* spp., *Cellulomonas* spp., *Bacillus* spp. and *Acetivibrio cellulolyticus* [11] (Table 1). Recently, Ejaz et al. [21,32] revealed the cellulolytic potential of *Bacillus aestuarii*, *Brevibacillus borstelensis* and *Aneurinibacillus thermoaerophilus* by using sugarcane bagasse as a substrate. For these new bacterial species that produce thermostable cellulases, further investigations are mandatory. Nowadays, most studies on thermostable cellulase production emphasize the optimization studies and development of new techniques to improve production rate such as the use of recombinant DNA technology and immobilized cells [21,22]. Isolation of thermophilic cellulase-producing bacteria have been reported from different environmental samples such as from compost sites [39], deep sea [40], marine plants [41], pond water [21] and soil [32]. Due to the increasing demand of thermostable enzymes, certain bioinformatics tools have been designed, which can predict protein stability and rigidity [7]. 

In addition, metagenomics has been introduced recently to discover novel cellulases [42]. Alvarez et al. [43] isolated a novel thermostable cellulase (CelE1) with unusual catalytic properties from sugarcane soil metagenome and studied its structure in detail. A canonical (β/α) 8 -barrel fold, comprised of eight β-sheets surrounded by eight α-helices, was observed. The opposite side of the active site contained an extra anti-parallel β-sheet, that is responsible for conformational stability. Two residues, a nucleophilic Glu^240^ and a proton donor Glu^152^, were identified as the catalytic acidic residues. Both of these residues were found 6.1 Å a part, which have compatible distances to retain a catalytic mechanism.

**Table 1:** Thermostable cellulases of different organisms.

Enzyme	Microorganism	Thermal Stability	Carbon Source	References
*bgl1*	*Thermoascus aurantiacus*	70% residual activity at 60 °C for 60 min	Cellobiose	[44]
*Egl* and *cbh1*	*Humicola grisea* var *thermoidea*	Stable at 60 °C for 10 min	Avicel and carboxymethyl cellulose	[45]
*Cbh3*	*Chaetomium thermophilum*	Half-life period 45 min at 70 °C	Microcrystalline cellulose and filter paper	[46]
CMCase	*Geobacillus* sp. HTA426	Stable at 50–70 °C for 5 h	Alkali-treated sugarcane bagasse, rice straw and water hyacinth	[47]

## 5. Application of Cellulase

In spite of more than three decades of utilization of cellulases in commercial sectors, this enzyme is still remained a topic of interest for both academic research as well as for industries [31,32]. Their wide scale applications in textile, animal food, pharmaceutical, detergent and paper processing industry ranked them at number two in global industrial enzyme market on the basis of business volume [7]. According to Pubmed.gov (as accessed on 17 June 2021), 26 reviews have been published on industrial application of cellulase in last 5 years which showed the importance of cellulase enzyme. The structure of cellulase and its mode of action have been deciphered extensively and, therefore, the term ‘cellulases’ now enjoys a workable definition. To glimpse the future, the question about the novel applications leads to numerous fascinating possibilities such as their utilization in food industry, which is a growing industrial and commercial sector.

### 5.1. Biofuels and Biorefineries

Cellulase hydrolyzes the biomass into simple sugars, either pentose or hexoses, which are then fermented to fuel or bioethanol. Cellulases are mainly involved in the bioconversion of renewable lignocellulosic biomass. Degradation of such biomass consists of 3 steps: (1) pretreatment of biomass, (2) saccharification in which enzymes are involved, and (3) fermentation. It is estimated that bioprocessing of biomass by cellulolytic microorganisms can reduce 40% of the process cost [10]. Currently, various countries have adopted policies regarding cellulosic ethanol and have set targets to shift the biomass resource from starchy or cane sugars to cellulose based materials [33,34]. Although a single microbial strain has yet to be available for the consolidated bioprocessing of plant materials, Chung et al. [48,49,50,51] reported that *Caldicelluloseruptor bescii* has the abilities to directly convert plant biomass in to bioethanol which shows the potential of ethanol production of this thermophilic bacteria which can be used in commercial sector for biomass conversion to bioethanol.

### 5.2. Paper and Pulp Industries

The use of cellulase in paper and pulp industry is still an emerging area. Pulping can be done by mechanical or biochemical methods. Mechanical process yields pulp with high stiffness, bulk and high content of fines whereas the use of cellulase in biochemical pulping results in 20–40% energy saving [52]. Kuhad et al. [5] reported that the use of cellulase offers many advantages as compare to xylanase such as improving the final brightness score and enhancing bleachability of softwood kraft pulp. Mostly, fungal cellulases especially *Aspergillus niger* and *Trichoderma reseei* are used for this purpose. Bacterial cellulase named CelB is also reported to improve paper properties [53]. 

### 5.3. Textile and Detergent Industry

The most popular and successful application of cellulases is in textile industry where these are used for jeans biostoning, biopolishing of fabrics and cotton and to improve appearance of fabrics. Household laundry detergents represents one of the most popular markets for enzyme sale accounting for 20–30%, with proteases and lipases as the main components along with cellulase [54,55]. For the detergent industry, cellulase should be compatible with alkaline conditions and with other ingredients of formulation and needs to be thermostable [39,40]. Particularly, alkaline cellulase improves the brightness of color and remove dirt from the fabric [56]. 

### 5.4. Animal Feed Industry

Other application of cellulase involves its use in animal feed industry. It can be utilized for the pretreatment of grain feed and agricultural silage to improve the nutritional value of animal feed [5]. Moreover, cellulases degrade the anti-nutritional components such as oligosaccharides, β-glucan, pectins, lignin, inulin, dextrins, cellulose and arabinoxylans which ultimately improve the nutritional value of feed and animal health [36,42,57].

### 5.5. Food Industry

**Figure 1:**
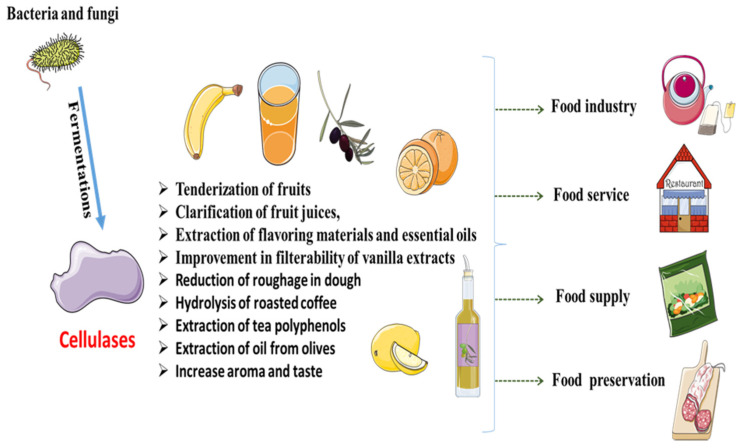
Cellulases application in food industry.

Food production and processing has become a major concern for mankind due to the climate change, urbanization and increasing population. It is necessary to produce food with better texture, flavor, color and to make it convenient for packing and consumption [58]. Enzymes can play a vital role to achieve many of the above targets in the commercial sector. However, the application of cellulases in food industries is yet to be acknowledged widely [59]. Cellulase from bacteria (*Paenibacillus* and *Bacillus*) and fungi (*Trichoderma* and *Aspergillus)* are potentially used in food industry [60]. There are many applications of cellulase in food industries, including tenderization of fruits, clarification of fruit juices, extraction of flavoring materials and essential oils and improvement in the filterability of vanilla extracts (Figure 1).

#### 5.5.1. Wine Industry

There are studies showing that application of cellulases to obtain good-quality wine. In nature, mostly sugars exist as hemicellulose and cellulose. Therefore, cellulosic biomass can be utilized for alcohol production. Cellulosic biomaterials can be converted to fermentable sugars by cellulase enzyme; sugars are then converted to alcohol by the yeast [58]. The use of cellulase in wine making offers many advantages such as quality and stability of wine, clarification, better color development, and improved maceration [58]. Cellulase reduces the wort viscosity as well. The aroma of wines could be enhanced by using β-glucosidases via hydrolyzing glycosylated precursors into glucose and aglycones [61]. An ethanol-tolerant endoglucanase from *A. niger* was isolated from wine fermenter by Xue et al. [62]; the enzyme was stable at a high temperature and acidic pH. 

#### 5.5.2. Olive Oil Extraction

Olive oil provides many health benefits and is very popular for domestic use. Cellulases are utilized for the extraction of oil from olives [5]. Their application results in less wastage, lower tendency to rancidity, increase in antioxidant components, better quality, and extraction yield [63]. The commercial enzyme preparation, Olivex (xylanase, cellulase and pectinase from *Aspergillus aculeatus*) was the first enzyme cocktail used to extract olive oil [52].

#### 5.5.3. Carotenoid Extraction

Carotenoids are the substances responsible for many plant colors from yellow to red. There is a continuously growing market for carotenoids for their commercial use. Usually a combination of pectinolytic and cellulolytic enzymes are used for extraction of carotenoid. Neagu et al. [64] used cellulase to extract carotenoids from tomatoes which have the potential to be used as coloring agents in the beverage and food industries. Cinar [65] extracted caotenoid pigments from carrot, sweet potato and orange peels by enzymatic hydrolysis in which pectinase and cellulase were used in combination. 

#### 5.5.4. Extraction of Phenolic Compound

Polyphenols are bioactive compounds that have been used to reduce the risk pf cancer and cardiovascular. Cellulases can play a key role in the extraction of phenolic compounds from grape pomace [66] and tea leaves [67]. Hai et al. [67] reported that cellulase addition to extract polyphenols from old tea leaves can improve the yield and the workers reported extraction of total of phenolic content of 85.05 mg GAE/g dry matter. 

#### 5.5.5. Baking

Baking is also affected by the pure form of cellulose, therefore, addition of cellulases converts the cellulose polymers to monomeric glucose; which results in reduction of roughage in dough [68]. In a report, purified cellulases from *Humicola insolens*, *Trichoderma reesi* and *Aspergillus niger* were used to reduce roughage in dough [68]. Cellulase can also be utilized for enzymatic hydrolysis of roasted coffee which uses less energy and is cheaper compared to thermal hydrolysis of coffee [69]. 

#### 5.5.6. Fruit Juice Industry

Raising health consciousness among the public resulted in greater demand of fruit juices. However, the presence of cellulosic polysaccharides hinders the traditional procedures of fruit juice extraction. Addition of cellulases during the fruit processing decreases the strength of the cell wall and also solubilizes the cellulosic polysaccharides which results in almost complete liquefaction. Cellulase reduces the viscosity of puree and nectar from fruits such as peach, pear, papaya, plum, mango and apricot and are also used for flavonoids extraction from seeds and flowers [61]. Fibers present in juices incur another issue for industries as being insoluble and denser; these can clog the manufacturing line and cause huge loss to industry [58]. As fibers are cellulosic in nature, addition of cellulases removes the fibers and makes the filtration of juices easier. Shariq and Sohail [70] were able to reduce turbidity of orange juice and obtain substantial decrease in acidity and viscosity by using multi-enzyme preparation made of cellulase and xylanase. The addition of cellulases is also known to increase aroma and taste of citrus fruits [71]. Food containing fibers can provide health benefits as well, such as reducing the risk of some types of cancer, diabetes, heart disease and also help to maintain a healthy body weight [72]. However, too much fibers in the diet can have some side effects on the health. Therefore, fibers should be consumed in moderation. 

Cellulases have great potential in improving the world food supply as it can play a significant role in food industry and preservation [73] for a better yield in food production. Moreover, we expect there to be further applications especially with advances in its purification, characterization, immobilization and production [55,56].

## 6. Enzyme Immobilization

Low activity in water environment, narrow pH range, thermal sensitivity, and the lack of recyclability of enzymes hinder their application in commercial sector. For large-scale industrial application, enzyme stability and reusability are required. Immobilization of enzymes in a gel or on a matrix renders them recyclable and hence the cost of the process can be reduced. Although, the term ‘immobilization’ has been referred to in literature since the twentieth century [74,75,76] research continues to have novel, stable and cost effective immobilization matrices. Various physical and chemical methods have been described for enzyme immobilization. These methods include cross linking, encapsulation, entrapment, covalent binding and adsorption [77]. Selection of the most appropriate support material and immobilization method strongly depend on the condition and type of the catalytic process [78].

In recent decades, most research studies showed that immobilized cellulase exhibits better structural stability, maintains high activity for a long time [79] and remains active at high pH as compared to the free enzyme [80,81,82]. The immobilized cellulases also have higher affinity to the substrates [83]. These properties present immobilized cellulase as an effective biocatalyst for cellulose bioconversion. Gordana et al. [83] immobilized cellulase with glutaraldehyde, a covalent cross-linking agent to produce cross-linked enzyme aggregates. Immobilization as cross-linked enzyme aggregates is highly attractive due to its operational stability, high activity, wide applicability, robustness, simplicity, easy recovery and has no necessity for very pure enzymes [83]. In another report, silica was used as a matrix for cellulase immobilization. The affinity of the enzymes to silica was also enhanced by modifying the matrices with polymers [84], for instance, cellulase exhibited higher affinity to the silica with polyamidoamine dendrimers than that of native silica [85]. Hartono et al. [86] prepared mesoporous silica and used it for immobilization of cellulase. Furthermore, mesoporous silica was modified with vinyl-cubic mesoporous silica to be used as a matrix for immobilized cellulase. This matrix provided greater enzyme stability and high enzyme activity. Ji et al. used magnetic nanoparticle to immobilize cellulase in order to improve enzyme stability and reusability for hydrolysis of bamboo biomass. 

In order to search for promising biocatalyst for biomass conversion, various studies have been conducted to assess the saccharification efficiency of immobilized cellulases. In this context, saccharification yield of 21% was reported with a recyclability of four cycles along with the retention of 38% activity [87]. Holocellulase produced by *A. niger* was covalently immobilized on magnetic enzyme–nanoparticle complexes for the saccharification of paddy straw and resulted in 375.39 mg gds^−1^ saccharification yield than free enzyme (339.99 mg gds^−1^) [88]. In another study, *Trichoderma reesei* cellulase was immobilized on silica and magnetic nanoparticles with immobilization efficiency of 76% and 85%, respectively. The nanobioconjugates exhibited an increase in thermal stability, pH, temperature optimum and V_max_ as compared with non-immobilized cellulase and immobilized cellulase retained its activity for five saccharification cycles [89]. Kamyar et al. [90] suggested that polyethylene glycol is nontoxic, biodegradable and biocompatible and can be used as a cross linker to immobilize cellulase. Pectinase (from *A. aculeatus*) and cellulase (from *T. reesei*) were co-immobilized on amino-functionalized magnetic nanoparticle to extract antioxidant from peels of banana, mango and orange. This enzymatic treatment released two fold higher free radical scavenging activity from the fruit peels compared to conventional solvent based extraction [91]. In another study, cellulase and pectinase were immobilized on amino-functionalized magnetic nanoparticle to extract lycopene from tomato peel [92].

## 7. Conclusions

The literature emphasizes that enzyme-based industries are gaining importance over the chemical-based industries due to process safety, low refining cost, high yield, efficient process control and friendly nature. Enzymes, particularly cellulases, have potential applications in paper, pharmaceutical, detergent and food industries. Thermostable cellulases have extensive use because of their stability at elevated temperature. The most promising application of cellulases nowadays are in the beverage, feed and food industries. We would like to emphasize again the importance of research on cellulases and their applications in the food industry. It is particularly important to cater to the diversified needs of food industry. 

## 8. Future Outlook

Although cellulases have widespread applications the cost of production impede their exploitation, particularly for cellulosic ethanol. Therefore, many laboratories are working on the strategies to reduce the production cost by employing waste materials as media components for enzyme production. Another area of research in focus will have novel immobilization matrices so that the catalytic process can be efficient and ultimately the cost can be reduced. The recent advancement in synthetic biology has opened a new arena for the development of chimeric cellulases. Improvements in the above areas of research will lead toward a sustainable use of resources, maximizing their use through cost-effective, low-energy and environment-friendly green bioprocesses.

## Data Availability

Not applicable.
